# Radiation in the Orthopedic Operating Room: What We Know, What We Do, and What Needs Attention

**DOI:** 10.7759/cureus.90911

**Published:** 2025-08-24

**Authors:** Muhammad A Hamid, Zubair Younis, Ariz Raza, Ali Tauseef, Mohd Amir Hasan Khan, Shahid Mir, Saif Abdulsattar, Nadia Rashid

**Affiliations:** 1 Trauma and Orthopaedics, East and North Hertfordshire Teaching NHS Trust, Stevenage, GBR; 2 Orthopaedics, The Royal Wolverhampton NHS Trust, Wolverhampton, GBR; 3 Trauma and Orthopaedics, University Hospitals Birmingham NHS Foundation Trust, Birmingham, GBR; 4 Anaesthesiology and Critical Care, University Hospitals Birmingham NHS Foundation Trust, Birmingham, GBR; 5 Trauma and Orthopaedics, Manchester University NHS Foundation Trust, Manchester, GBR; 6 Histopathology, Government Medical College, Srinagar, IND

**Keywords:** fluoroscopy, radiation, spine, surgery, trauma

## Abstract

Surgeons and operating room staff are frequently exposed to ionizing radiation, particularly during fluoroscopy-guided procedures in trauma and spine surgery. Despite established safety protocols, significant gaps remain in awareness, training, and adherence to best practices. While personal protective equipment such as lead aprons, thyroid shields, and lead glasses is effective, their inconsistent use leaves healthcare workers vulnerable. Recent studies raise particular concern over elevated cancer risks, including breast cancer among female orthopedic surgeons. Technological improvements and procedural modifications, such as pulsed fluoroscopy and optimized C-arm positioning, reduce exposure considerably but require wider adoption. Greater emphasis on education, adherence to regulations, and gender-specific research is essential to enhance radiation safety and protect the orthopedic workforce. This narrative review focuses on the risks associated with ionizing radiation use in orthopedic surgery, factors influencing exposure, as well as techniques and protective equipment that can be utilized to lower the risk.

## Introduction and background

Orthopedic surgeons, particularly those in trauma and spine specialties, often work in close proximity to radiation-emitting devices, such as C-arm fluoroscopes. Studies indicate that orthopedic surgeons may receive annual radiation doses that are close to or exceed recommended safety thresholds, especially when not wearing proper protective measures. Research also links personnel using fluoroscopy with increased risk of malignancy: of the thyroid, gonads, and solid organ cancers [1].

Despite this, research suggests that there is a considerable lack of knowledge around safety and practices of radiation safety within the operating room. A South African survey of orthopedic surgeons revealed that 73% surgeons had not received adequate training on radiation safety and 77% were not aware of the As Low as Reasonably Achievable (ALARA) principle [2]. ALARA is based on the principle that while the primary objective of radiography is producing imaging to aid in diagnosis, the radiation dose to the patient must be considered [3]. A study on orthopedic trainees in Ireland found low compliance with important techniques of reducing radiation exposure [4].

Sources of radiation exposure in orthopedic settings primarily include intraoperative fluoroscopy. Fluoroscopy is widely used for real-time imaging during procedures such as fracture reductions, internal fixations, and spinal surgeries, providing invaluable visual guidance but also exposing the surgical team and patient to ionizing radiation [1]. This narrative review was conducted by identifying relevant literature on radiation safety in orthopedic surgery. A targeted search was performed using databases including PubMed and Google Scholar for English-language articles published between 2000 and 2025. Keywords included “radiation exposure,” “orthopedic surgery,” “fluoroscopy,” “personal protective equipment,” and “dosimetry.” Additional guidelines and regulatory documents from the International Commission on Radiological Protection (ICRP), Ionizing Radiation Regulations (IRR), and Ionizing Radiation (Medical Exposure) Regulations (IR(ME)R) were reviewed. Studies were selected based on relevance to the topics of exposure risks, mitigation strategies, gender-specific concerns, and clinical best practices. As this is a narrative review, no formal risk-of-bias assessment or meta-analysis was performed.

## Review

The health risks associated with radiation exposure among orthopedic surgeons are significant. Prolonged or high-dose exposure has been linked to cataract formation, which is particularly concerning for healthcare workers given the radiosensitivity of the eye’s lens [5]. Thyroid dysfunction is another well-documented risk, as the thyroid gland is highly susceptible to ionizing radiation. More alarmingly, emerging evidence suggests higher incidences of certain cancers, including breast cancer, in female orthopedic surgeons [5]. These observations have raised concerns regarding the occupational hazards of continuous radiation exposure in orthopedic surgery. These are reflected in the strict guidelines and legislation in force for the use of ionizing radiation in medical settings. For instance, the Ionizing Radiation (Medical Exposure) Regulations (IR(ME)R) 2017 (amended in 2018) and the Ionizing Radiation Regulations 2017 (IRR 17) serve to regulate the use of IR in medical scenarios in the United Kingdom [6,7]. Furthermore, there is evidence to suggest that orthopedic surgeons themselves have inadequate knowledge of the risks they’re exposed to, and there are wide variations in the protective equipment used and in the practice of using dosimeters to monitor radiation exposure [8,9].

Biological effects of radiation exposure

1. Deterministic (non-stochastic) effects: These include effects such as hair loss and skin burns. Below a specific threshold dose, these deterministic effects do not occur [10]. These effects are worsened with additional exposures beyond the threshold [11].

2. Stochastic effects: Stochastic effects are random in nature, with the probability of occurrence proportional to the dose received. These effects are a function of dose without any minimum threshold [10]. These effects are more commonly associated with teratogenesis and carcinogenesis [12,13].

Radiation Safety Limits

International safety guidelines have been established to minimize occupational radiation exposure for healthcare workers. These guidelines set specific regulatory thresholds to ensure long-term safety. According to the International Commission on Radiological Protection (ICRP), the annual dose limits for occupational exposure are as follows: a whole-body dose of 20 mSv per year, averaged over five years, a maximum annual dose of 20 mSv for the lens of the eye, and 500 mSv per year for the skin and extremities. For pregnant workers, stricter guidelines are in place, with a recommended fetal exposure limit of less than 1 mSv throughout the pregnancy [14]. In addition to dose limits, the *time, distance, shielding* principle is a cornerstone of radiation safety [15].

Time

As radiation exposure is cumulative, time spent using fluoroscopy has a direct correlation with radiation exposure [16]. Fluoroscopy usage (especially live fluoroscopy) must be minimized as much as possible. Experienced surgeons use less radiation, and a skilful radiographer would use X-rays at appropriate angles and projections to prevent repeat shots [17]. This can be improved by employing educational programs aimed at orthopedic residents and faculty. This has been shown to positively impact the intra-operative radiation exposure to surgeons, staff, as well as patients [18,19]. It was seen that implementation of a standardized communication tool effectively reduced both patient as well as staff radiation exposure at a trauma center in Ireland [20]. Their study focused on improving communication between surgeons and radiographers so that radiation exposure to staff and patients could be minimized.

Distance

**Figure 1:**
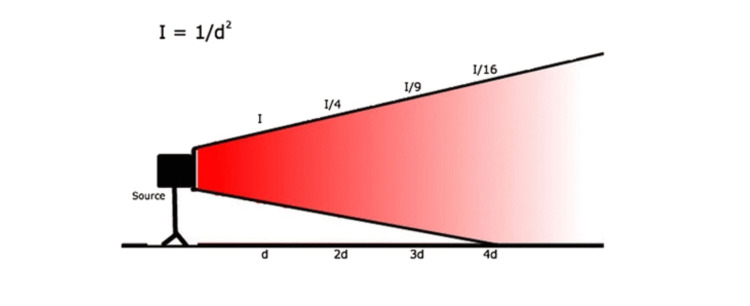
Inverse-square law. Inverse-square law: I = 1/*d*^2^, where *I* = magnitude of scatter and *d* = distance from the source. As the distance from the X-ray tube doubles, radiation exposure decreases to one-fourth. Image adapted from Kaplan et al. [4], licensed under CC BY 4.0.

Maintaining an appropriate distance from the radiation source is a critical component of radiation safety, as exposure levels decrease substantially with increasing distance. This principle is governed by the inverse square law, which states that doubling the distance from a radiation source results in a fourfold reduction in exposure [16]. Supporting this, one study demonstrated that standing just 20 cm away from the X-ray source reduced radiation exposure by approximately 73% [21]. As the distance increases further, the benefits become even more pronounced-at a distance of 2 meters, scatter radiation becomes virtually negligible, offering significant protection to operating room personnel (Figure 1) [22].

Radiation Safety Equipment: Knowledge and Practices

Protective equipment plays a central role in mitigating the risks of radiation exposure in orthopedic operating theaters. Personal protective equipment (PPE) is essential for all personnel, with shielding being a fundamental requirement. Shielding implies the use of physical barriers designed to absorb scattered radiation to some extent and prevent radiation penetration into soft tissues [23]. Inside the operating room, this is commonly accomplished by means of lead aprons and thyroid shields. These protect radio-sensitive organs of the body, starting from the thyroid to the gonads [24]. These aprons, which typically have a lead equivalence of 0.25 to 0.5 mm, are highly effective in reducing radiation absorption. Researchers have shown that mean radiation attenuation of up to 90% and 97% can be achieved with lead aprons of 0.25 and 0.5 mm thickness (lead or lead-free), respectively [25].

Despite this, there are concerns that standard lead aprons and vests do not adequately shield the upper outer quadrant of the breast, which is the site most affected by malignant change. In a study by Valone et al., it was shown that radiation exposure to the upper outer quadrant of the female breast is higher, especially when performing a cross-table lateral view. They recommended appropriate sizing of lead aprons to ensure a good fit, as smaller or larger aprons were associated with higher radiation exposure. They also recommended distancing the axilla from the patient and the X-ray tube [26]. In another study, researchers evaluated methods for reducing intraoperative radiation exposure to the breast and found that lead sleeves and axillary supplements significantly decreased radiation exposure to the upper outer quadrant of the breast when compared to a well-fitting lead vest alone [27].

In addition to body protection, protecting radiosensitive organs such as the thyroid and eyes is critical. Thyroid shields are crucial for protecting the highly radiosensitive thyroid gland, and lead gloves are recommended for surgeons who must place their hands in or near the radiation field during procedures [28,29]. Additionally, lead glasses provide a significant reduction in radiation exposure to the lens of the eye, lowering the risk of cataract formation [30]. In a study assessing the protective nature of lead glasses in hip surgeons taking fluoroscopy images of the hip and pelvis, researchers found that lead glasses decrease the dose to the eyes by 10 times [31].

Dosimetry

Radiation monitoring devices are another component of radiation safety. Dosimeters, which measure cumulative radiation exposure, are worn by staff members to track their exposure levels over time. Common types of dosimeters include film badges, thermoluminescent dosimeters (TLDs), and electronic personal dosimeters (EPDs) [32]. It is recommended ideally that two dosimeters should be worn, one at the collar outside lead shielding, and the other on the torso underneath lead shielding. If only one dosimeter is available, it must be worn at the collar outside lead shielding to give an estimate of radiation dose to the unprotected regions of the head and neck [33]. A study by Hurley et al. has shown that appropriately used radiation protection (lead aprons, thyroid shields, and lead glasses) mitigates more than 90% of ionizing radiation in orthopedic fluoroscopic procedures. During seated procedures, though, radiation exposure to the inner thigh was higher [32]. These devices, though, do not account for accurate assessment of radiation exposure to all body parts, especially the hands, which are often cited as foremost in the line of radiation during orthopedic surgery. Also, factors such as patient body mass index (BMI) and their impact on radiation exposure were not assessed. Another study on the use of real-time dosimetry during orthopedic surgery found a significant reduction in surgeons’ radiation exposure once they were able to monitor it in real time with a dosimeter [34].

Operational practices can significantly reduce radiation exposure during orthopedic procedures. Radiographers should prioritize optimizing fluoroscopy usage by utilizing low-dose settings whenever possible. Intermittent fluoroscopy, rather than continuous imaging, can substantially reduce exposure while still providing necessary visual guidance. In their study on 316 patients undergoing spinal interventions, Goodman et al. noted that a combination of pulsed and low-dose modes decreased average radiation exposure time by 56.7% [35]. Despite the benefits in terms of radiation exposure, these low-dose pulsed techniques produce images of lower quality, which limits adoption by surgeons.

**Figure 2:**
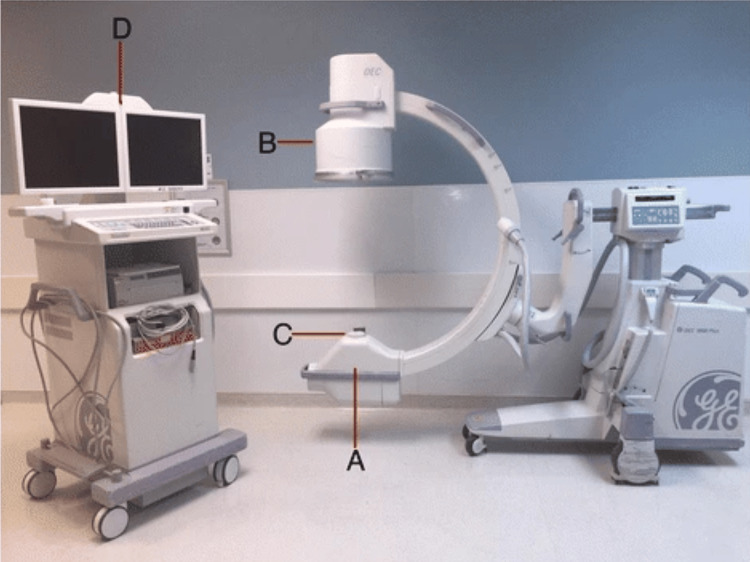
Mobile image intensifier unit: (A) X-ray tube; (B) image intensifier; (C) collimator; (D) display monitor. Mobile image intensifier. From Kaplan et al. [4]. Licensed under CC BY 4.0.

**Figure 3:**
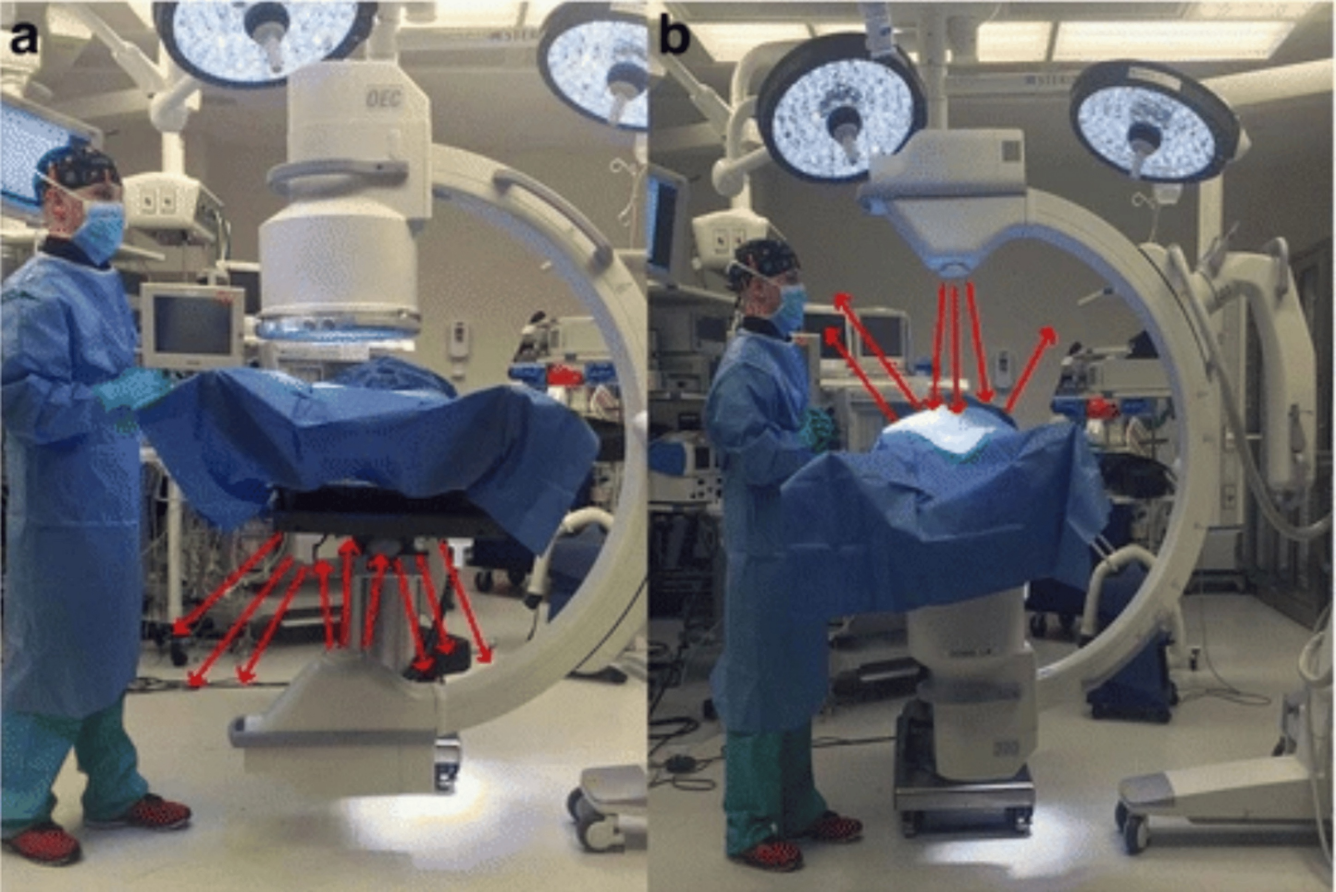
Radiation scatter pattern with the standard and "inverted" C-arm technique. Radiation scatter during fluoroscopy. From Kaplan et al. [4]. Licensed under CC BY 4.0.

Patient, C-arm, and personnel positioning are other critical factors: standing on the side of the image intensifier, rather than the X-ray source, can reduce exposure levels. A novel *inverted* C-arm technique employed during upper extremity orthopedic surgery found that the amount of exposure to both the patient as well as surgeon was significantly less in the *inverted* position of the fluoroscope [36]. Surgeons should also avoid placing their hands directly in the radiation field to minimize extremity exposure. Components of a typical image intensifier and a depiction of scatter radiation are shown in Figures 2-3.

Education and training are essential for promoting radiation safety awareness among healthcare workers. Regular radiation safety courses equip staff with the knowledge and skills needed to implement best practices. Organizational policies should support these efforts by establishing radiation safety committees, ensuring routine calibration and maintenance of imaging equipment, and mandating the use of dosimeters with regular reporting and review [37].

Avenues for Further Research

Despite advances in radiation safety, several areas warrant further investigation. Epidemiological studies are needed to better understand the long-term health consequences of occupational radiation exposure. The higher incidence of breast cancer among female orthopedic surgeons is particularly concerning and calls for large-scale cohort studies to identify potential causal relationships [26,38,39]. Similarly, more research is needed to elucidate the dose-response relationships associated with radiation-induced cataracts.

Technological innovation offers another promising avenue for improving radiation safety. The development of safer imaging technologies with reduced radiation output could significantly mitigate risks. Advancements in wearable protective gear, such as lighter and more ergonomic lead-free aprons, would enhance comfort and compliance among healthcare workers. Additionally, robotic-assisted systems could help minimize radiation exposure to personnel by allowing remote operation of imaging equipment. High implementation cost and limited evidence demonstrating clinical superiority of such systems presently hinder widespread adoption of the same in fields like spine surgery, where fluoroscopic guidance is key [40].

Gender-specific safety guidelines are an emerging area of interest. Given the unique risks faced by female healthcare workers, it is essential to evaluate whether current safety standards adequately address these concerns. Enhanced dosimetry devices that provide real-time feedback could also improve safety by enabling immediate adjustments to exposure levels. Integrating dosimetry data into electronic health records would facilitate better tracking and analysis of cumulative exposure.

Finally, radiation safety audits are a vital tool to assess adherence to radiation protection guidelines and ensure that staff who are directly involved in the use of medical radiation are appropriately trained, and their training refreshed at appropriate intervals to maintain proficiency [41].

## Conclusions

Radiation safety in orthopedic operating theaters is a critical concern. Clinical governance requires that personnel using ionizing radiation maintain high standards of protection for both patients and staff. While current protective measures and safety standards mitigate many risks, emerging evidence about long-term health consequences, especially among specific subgroups such as female surgeons, necessitates further research and innovation. By prioritizing education and investigating occupational health disparities, the orthopedic community can better safeguard its patients as well as practitioners while delivering high-quality care.
